# Benzodiazepine use and multidimensional health burden in severe psychiatric disorders: Impaired quality of life, metabolic comorbidities, and adverse effects in a large cross-sectional study

**DOI:** 10.1192/j.eurpsy.2025.10098

**Published:** 2025-09-03

**Authors:** Vincent Achour, Mélanie Faugere, Eloïse Maakaron, Jonathan Gavat, Guillaume Fond, Christophe Lançon, Théo Korchia

**Affiliations:** 1 Department of University Psychiatry, Sainte Marguerite University Hospital, Assistance Publique des Hôpitaux de Marseille, Marseille, France; 2 Assistance Publique des Hôpitaux de Marseille, Aix-Marseille University, UR3279: Health Service Research and Quality of Life Center – CEReSS, Marseille, France

**Keywords:** adverse effects, benzodiazepines, quality of life

## Abstract

**Background:**

Benzodiazepines (BZ) are widely prescribed to patients with severe mental illnesses, yet their long-term impact on global health remains underinvestigated. While their symptomatic benefits are acknowledged, data on their associations with quality of life (QoL), metabolic comorbidities, and side effects are limited.

**Methods:**

In this cross-sectional study, we analyzed clinical data from 1,248 patients with schizophrenia, bipolar disorder (BD), or major depressive disorder at a psychiatric center in Marseille, France. Associations between BZ use and key outcomes – including QoL (Short Form Health Survey [SF-36], EuroQol-5 Dimensions [EQ-5D], and Schizophrenia Quality of Life Questionnaire - 18 items [SQoL-18]), metabolic parameters, and treatment side effects (Udvalg for Kliniske Undersøgelser Side Effect Rating Scale [UKU scale]) – were examined using multivariate regression analyses.

**Results:**

BZ use was significantly associated with lower QoL scores on physical and mental health domains of the SF-36 (*p* < 0.001), increased impairment across EQ-5D dimensions, and reduced subjective well-being (SQoL-18, *p* = 0.043). BZ users also presented higher rates of obesity, diabetes, and metabolic syndrome (all *p* < 0.05). Furthermore, BZ use was independently associated with a higher burden of side effects across UKU subscales, particularly in the psychiatric domain (emotional blunting, anxiety, and depressive symptoms; *p* = 0.003).

**Conclusion:**

These findings suggest that BZ use in severe psychiatric disorders may be linked to a substantial multidimensional health burden, including reduced QoL, greater side effect profile, and increased metabolic risk. These results highlight the need for evaluation of long-term BZ use and the promotion of safer alternative treatments.

## Introduction

Schizophrenia (SZ), bipolar disorder (BD), and major depressive disorder (MDD) are severe psychiatric conditions that affect a substantial portion of the global population [1–3]. These disorders are characterized by significant disruptions in cognitive [4, 5], emotional [6], and behavioral functions [7]. The symptomatic burden of SZ severely impairs patients’ social and occupational functioning [8], contributing to long-term disability and a decrease in their overall quality of life (QoL) [9]. Although treatment typically involves antipsychotic medications [10] and psychotherapeutic interventions [11], many patients also receive adjunctive therapy with benzodiazepines (BZs) to manage comorbid symptoms, such as anxiety, agitation, and insomnia [12].

BZs are a class of pharmaceutical agents with a broad range of therapeutic actions, including anxiolytic, muscle-relaxing, and hypnotic properties. It is widely prescribed [13, 14] due to their rapid anxiolytic and sedative effects, particularly in acute settings [15, 16]. However, concerns regarding their long-term use have emerged [17]. Prolonged BZ administration has been linked to cognitive impairments [18], which are already frequent in SZ patients. Studies from the FondaMental Academic Centers of Expertise for Schizophrenia cohort suggest that patients stabilized on long-term BZ exhibit further cognitive decline [19]. In addition to cognitive effects, BZ use is associated with risks of dependence and withdrawal [20]. BZ use has also been linked to metabolic conditions; however, comprehensive data synthesizing this association remains limited [21–24].

Therefore, it is important to better understand how BZ use affects not only symptom management but also broader clinical variables, such as functional status, QoL, and the presence of metabolic comorbidities. While previous studies have examined BZ efficacy in symptom control [25], few have comprehensively assessed its impact on multidimensional clinical parameters, such as hypercholesterolemia or diabetes [23, 26], and long-term outcomes [27] in patients with major psychiatric disorders (MPDs).

The present study aims to fill this gap by exploring the effects of BZ use on a series of clinical and metabolic variables in patients diagnosed with SZ, BD, or MDD. Using a well-characterized clinical dataset, we will seek to identify correlations between BZ use and parameters, such as symptom severity, functional status, QoL, and the presence of metabolic comorbidities. Furthermore, this study will assess the potential long-term consequences of BZ use on the clinical course and prognosis of these patients, providing valuable insights into the risks and benefits of this common pharmacological intervention. This research will contribute to a better understanding of the complex interactions between pharmacotherapy, physical health, and QoL in psychiatric populations, potentially informing future clinical guidelines.

## Methods

### Study design and population

This study is a cross-sectional, observational investigation carried out at a specialized psychiatric center located in Marseille, France. It involved adult patients with confirmed diagnoses of SZ, MDD, or BD, based on the criteria outlined in the Diagnostic and Statistical Manual of Mental Disorders, 5th Edition. Participants were enrolled during psychiatric consultations or hospitalizations at the center between 2018 and 2023. The study utilized data from a monocentric database specifically developed to assess individuals receiving treatment for these psychiatric disorders. This dataset encompasses demographic, clinical, and biological variables, facilitating the exploration of associations between BZ use and multiple outcomes, such as symptom severity, functional capacity, QoL, and the prevalence of metabolic comorbidities.

### Inclusion and exclusion criteria

Participants eligible for inclusion in the study were adults aged 18–65 years with a primary diagnosis of SZ, MDD, or BD. All participants were required to be undergoing treatment or follow-up at the psychiatric center for these conditions and to have provided signed informed consent allowing the use of their clinical data for research purposes.

Exclusion criteria encompassed individuals who were pregnant or planning to become pregnant within 6 months of enrollment, as well as those with severe or unstable chronic diseases or neurological conditions, such as autoimmune disorders or cancer, that could potentially influence the study results. Additionally, individuals with severe cognitive impairments or acute psychiatric conditions that rendered them unable to complete clinical assessments were excluded.

### Data collection and clinical scales

Clinical data were gathered through structured medical consultations, reviews of medical records, and standardized interviews. BZ users were defined as patients currently prescribed a BZ at the time of clinical assessment, based on outpatient medical records and clinician-verified medication lists. Only patients with an active, regularly prescribed BZ treatment at the time of assessment were considered users. Occasional (pro re nata [PRN]) prescriptions were not included in the database. Sociodemographic information, including age, gender, employment status, and education level, was documented alongside psychiatric and somatic histories. Clinical scales were administered by trained healthcare professionals, and each patient completed the questionnaires under the supervision of a clinician to ensure proper understanding and accurate responses. Data quality was maintained through random data checks, personnel training, and supervised completion of questionnaires. All biological analyses were performed in a single laboratory to ensure consistency and accuracy, with threshold values determined according to local standards and the World Health Organization guidelines.

To comprehensively assess the participants’ clinical and functional profiles, a variety of standardized scales were utilized. The Global Assessment of Functioning (GAF) [28] was used to evaluate overall functional capacity and remission. Anxiety levels were measured using the State–Trait Anxiety Inventory [29], while treatment adherence was assessed with the Medication Adherence Rating Scale [30]. QoL was evaluated using the Short Form Health Survey (SF-36) [31], covering physical capacities, physical limitations, pain, perceived health, vitality, social functioning, emotional limitations, and mental health, with composite scores for physical and mental health calculated. The EuroQol-5 Dimensions (EQ-5D) [32] captured mobility, self-care, usual activities, pain/discomfort, anxiety/depression, and overall perceived health through a visual analog scale (EuroQol Visual Analog Scale [EQ-VAS]) [33]. Suicidal behaviors were screened using the Suicide Behaviors Questionnaire–Revised [34], while symptom severity and treatment response were assessed with the Clinical Global Impression Scale (CGI) [35]. Side effects were evaluated using the Udvalg for Kliniske Undersøgelser Side Effect Rating Scale (UKU) [36]. The Schizophrenia Quality of Life Questionnaire - 18 items (SQoL-18), a validated tool for assessing QoL across psychiatric conditions [37], was used for this study. The questionnaire includes 18 items describing 8 dimensions (psychological well-being, self-esteem, family relationships, relationships with friends, resilience, physical well-being, autonomy, and sentimental life). It also includes a total score (index). The eight dimensions and the index score range from 0 to 100; higher scores indicate a better QoL. The questionnaire had also been validated in bipolar and depressive disorders, allowing transdiagnostic analysis [38].

### Statistical analysis

All statistical analyses were conducted using SPSS software (version 20.0). Continuous variables were summarized using means and standard deviations, while categorical variables were described through frequency distributions. Comparisons between individuals with and without BZ use were performed using the chi-square test for categorical data, and Student’s *t*-test was employed for continuous variables. To explore factors associated with BZ use and various clinical or metabolic parameters, multiple regression analyses were carried out, adjusted for age, sex, educational level, antidepressants, chlorpromazine equivalent dose, current tobacco smoking, abdominal obesity, polypharmacy status, and diagnosis (SZ, MDD, and BD). Statistical significance was determined at a threshold of *p* < 0.05.

## Results

A detailed breakdown of the results is provided in Table 1.

**Table 1.** Factors associated with benzodiazepine (BZ) consumption

	Whole sample	No BZ	BZ	Univariate analysis	Multivariate analysis^a^	
	*N* = 1248 (100%)	*N* = 865 (69.3%)	*N* = 383 (30.7%)	(*p*)	Standardized betas	(*p*) adjusted
Diagnosis						
Schizophrenia	554 (44.4%)	394 (71.1%)	160 (28.9%)	-		
Major depressive disorder	533 (42.7%)	359 (67.4%)	174 (32.6%)	-		
Bipolar disorder	161 (12.9%)	12 (69.6%)	49 (30.4%)	0.403		
Sociodemographic						
Sex						
Men	723 (57.9%)	505 (58.4%)	218 (56.9%)	-		
Women	525 (42.1%)	360 (41.6%)	165 (43.1%)	0.629		
Age (years)	39.66 (14.33)	37.89 (14.32)	43.65 (13.56)	<0.001		
Working force status	243 (19.8%)	184 (21.8%)	59 (15.5%)	0.011		
Single	783 (63.8%)	556 (65.7%)	227 (59.6%)	0.038		
Education level (≥12 years)	555 (65.1%)	410 (69.5%)	145 (55.1%)	<0.001		
Education level (years)	12.51 (3.08)	12.81 (2.94)	11.84 (3.28)	<0.001		
Clinical characteristics						
Age at first episode (years)	24.43 (11.52)	23.54 (10.60)	26.54 (13.23)	0.012		
Disease duration (years)	12.39 (10.43)	11.32 (9.86)	14.90 (11.29)	0.001		
Lifetime suicide attempt (yes)	154 (25.3%)	107 (24.6%)	47 (27.0%)	0.536		
Clinical scales						
CDSS^b^ score	5.77 (5.56)	5.05 (5.01)	7.48 (6.40)	<0.001		
CDSS cutoff (≥6)	270 (42.4%)	170 (37.9%)	100 (53.2%)	<0.001		
GAF^c^	53.38 (15.61)	54.93 (16.03)	49.92 (14.07)	<0.001		
STAI-YA^d^	49.57 (13.88)	47.79 (13.90)	53.14 (13.16)	<0.001		
MARS^e^	6.17 (2.31)	6.11 (2.39)	6.31 (2.12)	0.181		
SBQ-R^f^	8.73 (5.10)	8.41 (4.92)	9.45 (5.43)	0.012		
CGI^g^	4.19 (1.19)	4.04 (1.21)	4.54 (1.07)	<0.001		
UKU^h^ I	5.60 (4.89)	5.19 (4.96)	6.42 (4.64)	0.003	0.178	0.011
UKU II	1.18 (1.55)	1.04 (1.42)	1.46 (1.76)	0.003	0.156	0.032
UKU III	2.82 (2.79)	2.48 (2.62)	3.49 (2.99)	<0.001	0.213	0.003
UKU IV	3.77 (4.16)	3.37 (3.82)	4.56 (4.67)	0.001	0.191	0.008
Quality of life scales						
SF–36^i^ physical functioning	72.10 (25.97)	76.61 (23.77)	62.01 (27.86)	<0.001	−0.210	0.003
SF–36 role physical	36.63 (39.67)	43.20 (41.47)	22.24 (30.99)	<0.001	−0.143	0.073
SF–36 bodily pain	56.79 (27.40)	61.08 (25.90)	47.40 (28.30)	<0.001	−0.146	0.063
SF–36 general health	45.35 (20.45)	48.81 (20.40)	47.40 (28.30)	<0.001	−0.261	0.001
SF–36 vitality	33.44 (20.51)	32.81 (20.88)	28.33 (18.78)	<0.001	−0.142	0.065
SF–36 social functioning	39.38 (25.16)	43.10 (25.91)	31.23 (21.35)	<0.001	−0.208	0.006
SF–36 role emotional	24.00 (35.15)	27.87 (37.58)	15.44 (27.32)	<0.001	−0.063	0.421
SF–36 mental health	39.14 (21.10)	42.98 (21.41)	30.76 (17.79)	<0.001	−0.284	<0.001
SF–36 physical health score	47.00 (13.02)	49.26 (12.91)	42.06 (11.87)	<0.001	−0.191	0.015
SF–36 mental health score	28.87 (13.84)	30.78 (14.70)	24.77 (10.74)	<0.001	−0.171	0.030
EQ–5D^j^ mobility	1.41 (0.81)	1.34 (0.72)	1.59 (0.96)	0.011	0.001	0.995
EQ–5D autonomy	1.22 (0.59)	1.19 (0.56)	1.29 (0.62)	0.152	−0.001	0.989
EQ–5D usual activities	2.04 (1.09)	1.96 (1.03)	2.22 (1.20)	0.040	0.015	0.869
EQ–5D pain/discomfort	1.98 (1.05)	1.83 (0.93)	2.30 (1.22)	<0.001	0.081	0.367
EQ–5D anxiety/depression	2.56 (1.23)	2.41 (1.17)	2.89 (1.29)	<0.001	0.133	0.147
EQ–5D VAS	56.48 (21.62)	59.24 (20.40)	50.77 (22.99)	<0.001	−0.137	0.178
SQoL–18^k^ psychological well-being	50.66 (28.73)	51.41 (28.22)	48.91 (29.85)	0.241	−0.077	0.236
SQoL–18 self-esteem	44.50 (30.72)	46.32 (30.24)	40.17 (31.48)	0.007	−0.066	0.298
SQoL–18 relationship with family	64.40 (29.30)	65.67 (27.76)	61.42 (32.51)	0.063	−0.041	0.515
SQoL–18 relationship with friends	48.65 (30.30)	49.63 (29.83)	46.29 (31.31)	0.134	−0.098	0.130
SQoL–18 resilience	53.73 (28.04)	54.66 (27.23)	51.55 (29.80)	0.140	−0.079	0.213
SQoL–18 physical well-being	40.49 (28.88)	42.03 (28.46)	36.90 (29.58)	0.014	−0.028	0.648
SQoL–18 autonomy	57.36 (28.73)	58.71 (27.67)	54.21 (30.88)	0.040	−0.165	0.009
SQoL–18 sentimental life	35.93 (32.10)	36.99 (31.71)	33.37 (32.95)	0.132	−0.121	0.062
SQoL–18 index	48.98 (19.61)	50.22 (19.22)	46.02 (20.26)	0.004	−0.129	0.043
Psychiatric comorbidities						
ADHD^l^	99 (7.9%)	77 (8.9%)	22 (5.8%)	0.058		
Anxiety						
Agoraphobia	207 (16.8%)	115 (13.5%)	92 (24.5%)	<0.001		
General anxiety disorder	411 (33.4%)	256 (30.0%)	155 (41.1%)	<0.001		
Panic disorder	257 (20.9%)	156 (18.3%)	101 (26.9%)	0.001		
Social anxiety disorder	212 (17.2%)	137 (16.1%)	75 (19.9%)	0.101		
PTSD^m^	70 (5.7%)	47 (5.5%)	23 (6.1%)	0.672		
Substance use (current)						
Tobacco smoking	639 (54.1%)	418 (51.2%)	221 (60.5%)	0.003		
Tobacco (packs per years)	14.44 (15.29)	12.98 (15.18)	17.67 (15.06)	<0.001		
Fagerström score	4.71 (2.97)	4.33 (3.06)	5.48 (2.64)	<0.001		
Cannabis consumption	222 (18.8%)	153 (18.6%)	69 (19.1%)	0.856		
Alcohol use disorder	205 (17.5%)	126 (15.5%)	79 (22.1%)	0.006		
Medications						
Chlorpromazine equivalent dose	407.84 (639.91)	369.13 (559.68)	495.27 (785.60)	0.005		
Antipsychotics (all)	648 (51.9%)	439 (50.8%)	209 (54.6%)	0.213		
Atypical antipsychotics	614 (49.2%)	426 (49.2%)	188 (49.1%)	0.958		
Typical antipsychotics	109 (8.7%)	45 (5.2%)	64 (16.7%)	<0.001		
Antipsychotics, long-acting	84 (6.7%)	62 (7.2%)	22 (5.7%)	0.355		
Antidepressants	606 (48.6%)	355 (41.0%)	251 (65.5%)	<0.001		
Mood stabilizers	119 (9.5%)	76 (8.8%)	43 (11.2%)	0.176		
Physical health						
Body mass index	25.64 (5.59)	25.46 (5.64)	26.05 (5.47)	0.086		
Total cholesterol (mM)	5.30 (5.71)	5.05 (1.12)	5.86 (10.04)	0.025		
hsCRP^n^ (mg/L)	2.19 (2.25)	2.03 (2.18)	2.56 (2.37)	0.001		
TSH^o^ (mIU/L)	2.24 (1.49)	2.32 (1.59)	2.07 (1.23)	0.007		
Prolactin (mIU/L)	467.15 (673.09)	451.31 (694.82)	501.65 (622.86)	0.276		
Vitamin D (nM)	58.58 (31.53)	59.61 (31.97)	56.25 (30.42)	0.120		
Vitamin B12 (pM)	346.04 (137.84)	342.40 (123.85)	354.64 (166.36)	0.287		
Vitamin B9 (folates, nM)	15.56 (8.30)	15.67 (8.41)	15.32 (8.05)	0.610		
Hypertension (diagnosed)	99 (8.0%)	59 (6.9%)	40 (10.5%)	0.030		
Diabetes (diagnosed)	52 (4.2%)	28 (3.3%)	24 (6.3%)	0.014		
Metabolic syndrome (point of care)						
I Hypertension	465 (37.5%)	318 (37.0%)	147 (38.7%)	0.577		
II Hyperglycemia	153 (12.6%)	90 (10.7%)	63 (16.8%)	0.003		
III Hypertriglyceridemia	271 (22.5%)	174 (21.0%)	97 (25.9%)	0.057		
IV Low HDL cholesterol	280 (23.6%)	194 (23.8%)	86 (23.2%)	0.823		
V Abdominal obesity	743 (62.1%)	487 (58.7%)	256 (69.6%)	<0.001		
Metabolic syndrome	269 (22.5%)	172 (20.8%)	97 (26.1%)	0.040		

a Adjusted for age, sex, educational level, antidepressants, chlorpromazine equivalent dose, current tobacco smoking, and abdominal obesity.

b Calgary Depression Scale for Schizophrenia.

c Global Assessment of Functioning.

d State–Trait Anxiety Inventory—YA form.

e Medication Adherence Rating Scale.

f Suicide Behaviors Questionnaire—Revised.

g Clinical Global Impression.

h Udvalg for Kliniske Undersøgelser.

i 36-Item Short Form Health Survey Questionnaire.

j EuroQol-5 Dimensions.

k Schizophrenia Quality of Life—18 items.

l Attention deficit and hyperactivity disorder.

m Post-traumatic stress disorder.

n High-sensitivity C-reactive protein.

o Thyroid-stimulating hormone.

### Sample characteristics

The study included 1,248 participants with a mean age of 39.66 years (standard deviation [SD] = 14.33), categorized into three diagnostic groups: SZ (*n* = 554, 44.4%), MDD (*n* = 533, 42.7%), and BD (*n* = 161, 12.9%). The mean education level was 12.51 years (SD = 3.08). The mean age at first episode was 24.43 years (SD = 11.52), and the mean duration of illness was 12.39 years (SD = 10.43). BZs were prescribed within the range of approved therapeutic doses [39], corresponding to 5–40 mg/day of diazepam equivalents. In our sample, the most used molecules were alprazolam, diazepam, and oxazepam.

Regarding clinical symptom severity, the mean CDSS score was 5.77 (SD = 5.56), and the CGI severity score averaged 4.19 (SD = 1.19), indicating mild-to-moderate depressive and overall symptom severity. The mean Global Assessment of Functioning (GAF) score was 53.38 (SD = 15.61), reflecting moderate functional impairment.

For QoL, the SF-36 physical health score was 47.00 (SD = 13.02), and the SF-36 mental health score was 28.87 (SD = 13.84). On the EQ-5D scale, participants reported significant impairments across multiple dimensions, including mobility (1.41, SD = 0.81), usual activities (2.04, SD = 1.09), and anxiety/depression (2.56, SD = 1.23). The mean EQ-VAS score was 56.48 (SD = 21.62), reflecting a moderate perception of overall health. The mean SQoL-18 index score was 48.98 (SD = 19.61).

Mean age was significantly higher in the BZ group (43.65 years, SD = 13.55) compared to the non-BZ group (37.89 years, SD = 14.32; *p* < 0.001), suggesting that BZ use is more prevalent among older patients. Similarly, the mean level of education was significantly lower in the BZ group (11.84 years, SD = 3.28) than in the non-BZ group (12.81 years, SD = 2.94; *p* < 0.001).

The mean age at first episode was significantly later in the BZ group (26.54 years, SD = 13.23) compared to the non-BZ group (23.54 years, SD = 10.60; *p* = 0.012). Likewise, the mean duration of illness was longer in the BZ group (14.90 years, SD = 11.29) than in the non-BZ group (11.31 years, SD = 9.86; *p* = 0.001).

### Quality of life

Analysis of the QoL scales revealed significant differences between patients using BZ and those who were not. For the SF-36, physical health scores were lower in the BZ group (42.06, SD = 11.87) compared to the non-BZ group (49.26, SD = 12.91; *p* = 0.015). Similarly, mental health scores were reduced in the BZ group (24.77, SD = 10.74) compared to the non-BZ group (30.78, SD = 14.70; *p* = 0.030). These results indicate a global and multidimensional deterioration in perceived QoL among patients exposed to BZ. In multivariate analyses of the SF-36 subscales, BZ use was independently associated with lower QoL scores across several dimensions. The strongest associations were observed for general health (*B* = −0.261, *p* = 0.001), physical functioning (*B* = −0.210, *p* = 0.003), and social functioning (*B* = −0.208, *p* = 0.006). Mental and physical health scores were also significantly lower (*B* = −0.171, *p* = 0.030; *B* = −0.191, *p* = 0.015), reflecting the multidimensional burden of BZ use. Antidepressant use was significantly associated with lower scores in vitality (*B* = −0.217, *p* = 0.011), social functioning (*B* = −0.226, *p* = 0.007), and emotional role limitations (*B* = −0.229, *p* = 0.008). Age (*B* = −0.184, *p* = 0.046) and abdominal obesity (*B* = −0.161, *p* = 0.034) were both negatively associated with physical functioning, while higher education emerged as a protective factor (*B* = 0.140, *p* = 0.046).

In the multivariate analysis of the SQoL-18 index, BZ use was significantly associated with lower QoL scores (*B* = −0.129, *p* = 0.043). SQoL-18 Autonomy dimension was also significant (*B* = −0.165, *p* = 0.009).

Findings from the EQ-5D confirmed this trend. In univariate analysis, patients in the BZ group reported significantly higher levels of impairment in mobility (1.59, SD = 0.96 vs. 1.34, SD = 0.72; *p* = 0.011), usual activities (2.22, SD = 1.20 vs. 1.96, SD = 1.03; *p* = 0.040), pain/discomfort (2.30, SD = 1.22 vs. 1.83, SD = 0.93; *p* < 0.001), and anxiety/depression (2.89, SD = 1.29 vs. 2.41, SD = 1.17; *p* < 0.001) compared to non-BZ users, reflecting greater functional impairment and a negative perception of health status. Global health assessment measured by the EQ-VAS was also significantly lower in the BZ group (50.77, SD = 22.99) compared to the non-BZ group (59.24, SD = 20.40; *p* < 0.001). Unfortunately, these results did not hold in multivariate analyses.

### Metabolic comorbidities

BZ use was significantly associated with several metabolic comorbidities in univariate analyses, including diagnosed diabetes (*χ*^2^ = 6.03, *p* = 0.014), point-of-care hyperglycemia (*χ*^2^ = 8.78, *p* = 0.003), metabolic syndrome (*χ*^2^ = 4.21, *p* = 0.040), and abdominal obesity (*χ*^2^ = 12.67, *p* < 0.001). A significant association was also found with a history of hypertension (*χ*^2^ = 4.68, *p* = 0.030). Although not reaching statistical significance, trends were observed toward higher rates of statin use (*χ*^2^ = 3.29, *p* = 0.070) and hypertriglyceridemia (*χ*^2^ = 3.61, *p* = 0.057) in the BZ group.

### Adverse effects

Regarding adverse effects, results from the UKU scale demonstrated a significantly higher prevalence of side effects among patients using BZs. The subscales UKU I (psychic side effects: *p* = 0.003; mean difference = 1.23), UKU II (neurological side effects: *p* = 0.003; mean difference = 0.42), UKU III (autonomic symptoms: *p* < 0.001; mean difference = 1.01), and UKU IV (other side effects: *p* = 0.001; mean difference = 1.19) indicated a significantly higher frequency of adverse effects in the BZ group.

Furthermore, the multivariate analyses revealed a consistent and significant association between BZ use and increased side-effect scores across the various UKU subscales. For psychic side effects (UKU I), BZ use was associated with significantly higher scores (*B* = 0.178, *p* = 0.011), indicating a greater prevalence of symptoms, such as fatigue, dizziness, and somatic complaints.

In the neurological domain (UKU II), patients using BZs reported higher scores (*B* = 0.156, *p* = 0.032), reflecting increased coordination problems, sedation, and memory impairments.

The strongest association was observed in the autonomic domain (UKU III), where BZ use significantly increased scores (*B* = 0.213, *p* = 0.003). Patients on BZs reported higher rates of autonomic adverse effects, such as orthostatic dizziness, palpitations, increased salivation, and accommodation disturbances.

For other side effects (UKU IV), BZ use was also significantly associated with increased scores (*B* = 0.191, *p* = 0.008).

## Discussion

This study examined the impact of BZ use on QoL, symptom severity, and adverse effects in patients with SZ, BD, and MDD. Our findings indicate that BZ use is consistently associated with lower QoL, as evidenced by reduced scores in the SF-36 and SQoL-18 indices. Additionally, BZ use was linked to greater functional impairment in the EQ-5D and increased prevalence of adverse effects across all UKU subscales. Regarding overall health, BZ prescription was also associated with increased rates of medical comorbidities, including diabetes, obesity, and hypertension. These results suggest that while BZ may be prescribed for symptomatic relief, its long-term impact on well-being and treatment outcomes warrants careful consideration. Although BZ-related impairment might differ across diagnostic categories, the inclusion of diagnosis as a covariate in our multivariate models did not substantially alter the observed associations. The absence of significant interactions between diagnosis and BZ use supports the hypothesis of a transdiagnostic effect.

### Quality of life

Regarding QoL, numerous studies have established a link between antipsychotics and improved SF-36 and SQoL-18 scores across SZ, BD, and MDD [40]. However, available data on BZs remain scarce, and to our knowledge, our study is the first to specifically evaluate their impact on these parameters, with a sample including all three MPDs. A mean SF-36 mental health score of 25 reflects a profound psychological impairment, as healthy adults generally score above 70 [41]. EQ-VAS scores around 50 in our sample also highlight a notably reduced health perception compared to typical values above 70 [42]. Regarding QoL, few studies have used the SF-36 and SQoL-18 scales to compare differences between patient groups with MPDs. In a study conducted on bipolar patients, where QoL was assessed using the GAF scale, the authors found that BZ prescription was associated with a deterioration in QoL [43].

### Adverse effects

Regarding symptom severity and adverse effects, the UKU scale scores reflect the detrimental impact of BZ use on patients’ health, revealing a wide range of adverse effects. These include neuropsychiatric symptoms, such as emotional blunting, depressive mood, cognitive impairment, and sedation, as well as somatic symptoms like asthenia and appetite changes. These phenomena appear to perpetuate the disturbances also highlighted in our study, particularly regarding QoL and cardiometabolic health, creating a cycle of reciprocal influence. Our study’s findings reinforce trends observed in available studies, although these remain limited, and there is a significant lack of synthesized data. In a cohort of patients with MDD, Rizvi et al. found a significant association between anhedonia and BZ use. Furthermore, early works on the database we used in our study showed that the psychic side effects were the most significant predictors of altered QoL [44, 45]. As previously noted, while numerous studies have examined the impact of antipsychotic medications on adverse effects, the specific role of BZs remains unclear and underexplored [46], and our study is the first to our knowledge using the UKU scale to determine the burden of BZ use in our population.

### General health implications

Given that cardiac [47, 48] and metabolic [49, 50] complications are among the leading causes of morbidity and mortality in patients with severe psychiatric disorders; it is essential to highlight the significant findings of our study regarding these parameters. Our results, consistent with the limited existing literature [51], suggest that BZ prescription is associated with an increased risk of diabetes, obesity, and metabolic syndrome. These findings call for heightened clinical vigilance regarding cardiovascular risk factors in patients treated with BZs.

### Clinical implications

Although BZ can alleviate residual symptoms of MPDs, as well as various anxiety manifestations, our study suggests that these agents are linked to a deterioration in patients’ QoL and multimodal adverse effects. These observations call for a reassessment of their role in the therapeutic arsenal of mental health care in favor of alternative interventions. Given these findings, it appears crucial to promote alternatives to BZs for the management of residual symptoms in severe psychiatric disorders. Behavioral psychotherapeutic interventions, such as Mindfulness-Based Cognitive Therapy [52], have demonstrated efficacy in reducing anxiety and inner tension. These approaches can be easily implemented through psychoeducational sessions and are increasingly supported by digital health tools and mobile applications [53]. Pharmacological alternatives to BZs also exist and deserve greater clinical attention. Antihistamines, certain atypical antipsychotics, and agents such as buspirone have overlapping indications with BZs but offer a more favorable profile in terms of misuse potential and dependency risk [54]. Considering our findings, it appears that the international consensus regarding BZ use has historically been too permissive, and stricter regulation of both prescription practices and medication dispensing could be considered to mitigate the inherent risks associated with this drug class. Recent recommendations from the World Health Organization on substance use disorders are aligned with this perspective [55].

### Strengths and limitations

This study presents several strengths that enhance the reliability and relevance of its findings. First, the large sample size (*n* = 1,248) provides substantial statistical power, allowing for robust analyses of BZ use across three MPDs. Second, the inclusion of multiple validated scales – such as SF-36, EQ-5D, UKU, and SQoL-18 – ensures a comprehensive assessment of QoL, functional status, and adverse effects, reinforcing the validity of the observed associations. Additionally, the study benefits from a clinically diverse population, covering a broad spectrum of psychiatric symptoms and treatment regimens, thereby improving the generalizability of the findings to real-world psychiatric care.

However, several limitations must be acknowledged. Due to the cross-sectional design, no causal inference can be drawn. While BZ users consistently exhibited poorer QoL, greater functional impairment, a higher burden of side effects, and increased rates of metabolic comorbidities, it remains unclear whether these outcomes are a consequence of BZ exposure or reflect an underlying greater clinical severity at the time of prescription. Although residual confounding and reverse causality cannot be fully excluded – particularly from unmeasured factors, such as treatment resistance or psychiatric comorbidities – the consistency and convergence of associations across independent clinical dimensions, even after adjustment for psychotropic polypharmacy, suggest a meaningful and clinically relevant signal. We also explored the inclusion of a clinical severity proxy (CGI–Severity), available in a subset of patients. However, its incorporation substantially reduced the sample size and did not materially change the results. Given its partial availability and interpretative ambiguity in a cross-sectional framework, we chose not to retain it in the final models.

To ensure a more homogeneous sample of continuous users, we deliberately excluded patients with “as-needed” (PRN) prescriptions. While this design choice improves interpretability, it also limits the ability to assess cumulative exposure or treatment duration, as longitudinal medication histories were not consistently available. Similarly, dosage data were not standardized across the cohort, precluding the evaluation of dose–response relationships. These limitations underscore the need for prospective studies with detailed pharmacological monitoring.

Additionally, this study was conducted in a single academic psychiatric center, which may limit the generalizability of the findings to other care settings with different treatment protocols and prescribing practices. QoL and side effects were assessed using self-reported measures, which may be subject to recall bias or subjective interpretation, despite the use of validated and widely used tools.

To interpret these findings within a coherent conceptual framework, we propose that BZ use may act both as a proxy for clinical complexity (e.g., persistent symptoms, comorbid anxiety, and treatment resistance) and as a potential contributor to functional decline via known pharmacological mechanisms, including sedation, emotional blunting, and cognitive dulling. This dual role may contribute to a self-reinforcing clinical loop, whereby functional impairment increases reliance on BZ prescriptions, which in turn perpetuate or exacerbate decline. Although this model remains hypothetical at this stage, it offers a plausible explanation of our findings while acknowledging the design limitations.

Our results call for critical reflection on BZ prescribing practices in severe psychiatric disorders. They emphasize the importance of individualized, risk–benefit-based decision-making, and the development of alternative strategies – both pharmacological and psychotherapeutic – with better-documented long-term safety profiles. Future longitudinal studies integrating objective measures (e.g., cognitive function, functional trajectories, and neuroimaging) will be essential to elucidate the causal pathways involved and to guide clinical recommendations.

## Abbreviations

BD: bipolar disorder
BZ: benzodiazepines
CDSS: Calgary Depression Scale for Schizophrenia
CGI: Clinical Global Impression Scale
DSM-5: Diagnostic and Statistical Manual of Mental Disorders
EQ-5D: EuroQol-5 Dimensions
EQ-VAS: EuroQol Visual Analog Scale
ESRS: Extrapyramidal Symptom Rating Scale
GABA: Gamma-aminobutyric acid
GAF: Global Assessment of Functioning
MARS: Medication Adherence Rating Scale
MDD: major depressive disorder
MPD: major psychiatric disorders
PRN: pro re nata
SBQ-R: Suicide Behaviors Questionnaire–Revised
SF-36: Short Form Health Survey
STAI-YA: State–Trait Anxiety Inventory
SZ: schizophrenia
UKU: Udvalg for Kliniske Undersøgelser Side Effect Rating Scale
